# Mastering your fellowship: Part 4, 2024

**DOI:** 10.4102/safp.v66i1.5941

**Published:** 2024-05-13

**Authors:** Mergan Naidoo, Klaus von Pressentin, Andrew Ross, Selvandran Rangiah, Ts’epo Motsohi, Tabitha Mathose

**Affiliations:** 1 Department of Family Medicine, College of Health Sciences, University of KwaZulu-Natal, Durban, South Africa; 2 Department of Family, Community and Emergency Care, Faculty of Health Sciences, University of Cape Town, Cape Town, South Africa; 3 Department of Family Medicine, Faculty of Medicine, University of KwaZulu-Natal, Durban, South Africa; 4 Department of Family Medicine, Faculty of Health Science, University of KwaZulu-Natal, Durban, South Africa; 5 Department of Family Medicine and Primary Care, Faculty of Health Sciences, Stellenbosch University, Cape Town, South Africa

**Keywords:** family physicians, FCFP (SA) examination, family medicine registrars, postgraduate training, national exit examination, mental health

## Abstract

The ‘Mastering your Fellowship’ series provides examples of the question format encountered in the written and clinical examinations for the Fellowship of the College of Family Physicians of South Africa (FCFP [SA]) examination. The series is aimed at helping family medicine registrars prepare for this examination.

This section in the South African Family Practice journal is aimed at helping registrars prepare for the Fellowship of the College of Family Physicians of South Africa (FCFP [SA]) examination. It will provide examples of the question formats encountered in the written examination: a multiple choice question (MCQ) in the form of a single best answer (SBA – Type A) or extended matching question (EMQ – Type R); short answer questions (SAQs), questions based on the critical reading of a journal (evidence-based medicine) and an example of an objectively structured clinical examination (OSCE) question. Each question type is presented based on the FCFP blueprint and the key learning outcomes of the FCFP programme. The MCQs are based on the 10 clinical domains of family medicine, and the SAQs will be aligned with the 5 national unit standards. The critical reading section will include evidence-based medicine and primary care research methods.

This edition is based on unit standard one (critically reviewing new evidence and applying the evidence in practice, leadership and governance), unit standard two (evaluate and manage a patient according to the biopsychosocial approach) and unit standard five (conduct all aspects of healthcare in an ethical, legal and professional manner). The domain covered in this edition is mental health. We suggest you attempt to answer the questions (by yourself or with peers or supervisors) before finding the model answers online: http://www.safpj.co.za/.

Please visit the Colleges of Medicine website for guidelines on the fellowship examination: https://www.cmsa.co.za/view_exam.aspx?QualificationID=9.

We are keen to hear about how this series assists registrars and their supervisors prepare for the FCFP (SA) examination. Please email us (naidoom@ukzn.ac.za) your feedback and suggestions.

## Multiple choice question (MCQ): Single best answer

A 30-year-old woman presents to the emergency centre with chest pain and claims to have had an angiogram and stent inserted 4 years ago. She also states that she has cancer and was receiving chemotherapy but defaulted treatment. The doctors manage her as an acute coronary syndrome and admit her to the high care unit, but later discover that none of her assertions are correct. Examination findings and investigations are all normal. Which of the following is the most appropriate next step after appropriate counselling?

- Arrange a family conference.
- Certify her under the *Mental Health Care Act*.
- Discharge and refer her to a psychiatrist.
- Refer her to a psychologist.
- Refer her to the social worker.

Correct answer: b)

### Discussion

Factitious disorder, commonly known as Munchausen’s syndrome, is a mental health condition characterised by the fabrication or exaggeration of physical or psychological symptoms with the primary goal of assuming the sick role. This condition falls under the ‘Somatic Symptom and Related Disorders’ category in the Diagnostic and Statistical Manual of Mental Disorders, Fifth Edition (DSM-5).

In the case of the 30-year-old woman presenting with chest pain and false medical history, including claims of having undergone an angiogram and stent insertion, as well as receiving cancer treatment, the presentation strongly suggests a factitious disorder. It is essential to recognise that individuals with factitious disorder may go to great lengths to maintain the appearance of illness, including causing self-harm to support their claims. Given the potential for harm to herself, especially if she lacks insight into her condition, the most appropriate next step after appropriate counselling is to certify her under the *Mental Health Care Act* (MHCA). This action is necessary to ensure that she receives the appropriate psychiatric evaluation and treatment for her condition. Lack of insight into her condition further raises the risk of self-harm, emphasising the importance of prompt psychiatric evaluation and intervention.

The MHCA in South Africa, initially enacted in 2002 and amended in 2004, underwent further revisions in 2016 to strengthen the protection and promotion of the rights of individuals with mental health conditions. The amended MHCA provides an updated legal framework for the assessment, treatment and care of individuals with mental illness, ensuring their rights to dignity, autonomy and access to appropriate mental health services. Key provisions of the amended MHCA include the following:

#### Voluntary and involuntary admission

The amended MHCA elaborates on procedures for both voluntary and involuntary admissions to mental health facilities. It specifies criteria for involuntary admissions, such as a risk of harm to oneself or others due to mental illness. It introduces additional safeguards to protect the rights of individuals undergoing involuntary and assisted admission.

#### Capacity assessment

The amended MHCA underscores the importance of capacity assessment to determine individuals’ ability to make informed decisions about their mental healthcare and treatment. It provides detailed guidelines for assessing capacity and appointing substitute decision-makers when necessary, incorporating best practices and international standards.

#### Treatment and care

The amended MHCA reaffirms principles for the treatment and care of individuals with mental illness, emphasising the provision of evidence-based interventions, respect for cultural and religious beliefs and protection from abuse and neglect. It underscores the importance of a multidisciplinary mental healthcare approach involving primary care nurses or physicians, psychiatrists, psychologists, social workers and other mental health professionals. It promotes collaboration between mental health services and other sectors.

#### Rights and protections

The amended MHCA strengthens the rights of individuals with mental illness, including the right to dignity, privacy, confidentiality and freedom from discrimination. It reinforces prohibitions against using restraints and seclusion except in exceptional circumstances and with appropriate safeguards. The amended MHCA also enhances mechanisms for addressing complaints and grievances related to mental healthcare, ensuring greater accountability and transparency.

#### Community-based care

The amended MHCA underscores the importance of community-based mental health services, including outreach programs, community mental health centres and supported housing initiatives. It emphasises the integration of mental health services with other sectors, such as housing, employment and education, to promote holistic and integrated care for individuals with mental illness.

Certifying the patient under the MHCA allows mental health professionals to conduct a comprehensive assessment of her mental health status and determine the appropriate course of treatment. It also enables the provision of necessary interventions to address any underlying mental health issues and mitigate the risk of self-harm. Collaborating with other healthcare professionals, including psychiatrists, psychologists and social workers, ensures a multidisciplinary approach to her care, focusing on her well-being and safety.

In summary, factitious disorder is a serious mental health condition characterised by the fabrication or exaggeration of symptoms, often leading to unnecessary medical interventions. Certifying individuals under the MHCA is essential in cases of factitious disorder, especially when there are concerns about potential harm to oneself, lack of insight and the need for psychiatric evaluation and intervention. This ensures that patients receive the appropriate care and support to address their underlying mental health condition and minimise the risk of self-harm.

**Further reading**

- Republic of South Africa. Mental Health Care Act No. 17 of 2002, as amended. 2016.
- American Psychiatric Association. Diagnostic and statistical manual of mental disorders (5th ed.). Arlington, VA: American Psychiatric Publishing; 2013.

## Short answer question: The family physician’s role as a leader of clinical governance

At your district hospital (DH), you have many repeat admissions of poorly managed psychiatric patients. Patients are admitted when psychotic, treated until no longer psychotic and then referred back to the community health centre for ongoing management. There are always 3–4 psychiatric patients in the general male ward. These patients are disruptive and often stay longer than the 72-h observation period as the regional referral hospital is always full. Many of these patients have substance-induced psychosis.

1. As the family physician based at the hospital, what areas of concern arise from the scenario that could contribute to this problem? List two areas of concern under each heading: health system, patient and community factors. (6 marks)
2. Several clinical governance and leadership activities could be initiated to investigate and manage this issue. List four activities along with an appropriate explanation using the headings provided (Table 1a). (8 marks)
3. As a senior clinician, you want to develop a better relationship with the regional hospital to manage psychotic patients who present to your hospital. Outline a plan of how you would achieve this in point form. (5 marks)
4. Following a newspaper report about a psychiatric patient found dead on the grounds of a hospital, the chief executive officer (CEO) asks you to develop a protocol for the management of psychotic patients. In bullet form, list six issues that should form part of this protocol that would improve patient safety. (6 marks)

**TABLE 1a:** Clinical governance activity and outcome (linked to question 2).

Clinical governance activity	What do you hope to achieve with this activity?
	
	
	
	
	

**Total 25**

*Suggested answers*

1. **As the family physician based at the hospital, what areas of concern arise from the scenario that could contribute to this problem? List two areas of concern under each heading: health system, patient and community factors. (6 marks)**
  - Health system factors:
    ■ Staffing (hospital and psychiatric clinic)
    ■ No protocols
    ■ Infrastructure
    ■ Supplies (medication)
    ■ Limited support from regional hospital
    ■ Poor coordination of service between clinics, district and regional hospitals
    ■ Poor interdisciplinary care
    ■ Suboptimal management and review at the clinic
    ■ Lack of knowledge about management (doctors and nurses)
  - Patient factors:
    ■ Adherence
    ■ Psychosocial support at home
    ■ Education and insight into the condition and other factors influencing the capacity for self-management
    ■ Substance abuse
  - Community factors:
    ■ Socioeconomic conditions, including education levels and employment opportunities
    ■ Intersectoral collaboration
    ■ Stigma
2. **Several clinical governance and leadership activities could be initiated to investigate and manage this issue. List four activities along with an appropriate explanation using the headings provided (Table 1b). (8 marks)**
3. **As a senior clinician, you want to develop a better relationship with the regional hospital to manage psychotic patients who present to your hospital. Outline a plan of how you would achieve this in point form. (5 marks)**
  - Meet with the CEO and senior management at your DH to get their support and buy-in.
  - Meeting with the head of the department of psychiatry at the regional hospital (RH) to set up the following activities:
    ■ Outreach visits and in-reach placement.
    ■ Involve the RH’s psychiatric staff in your hospital’s morbidity and mortality meetings and quality improvement projects.
    ■ Involve RH psychiatric staff in the training program at your DH.
  - Develop joint strategies to improve resources at DH and RH (district office, department of health) beds, staff, etc. This would involve a review of the admissions at the DH, number of beds available at the DH, staff complement at the DH and a motivation to the district office/head office for the development of a plan to address these issues.
4. **Following a newspaper report about a psychiatric patient found dead on the grounds of a hospital, the CEO asks you to develop a protocol for the management of psychotic patients. In bullet form, list six issues that should form part of this protocol which would improve patient safety. (6 marks)**

**TABLE 1b:** Clinical governance activity and outcome (linked to question 2’s suggested answer).

Clinical governance activity	What do you hope to achieve with this activity?
Audit and feedback	Assess the quality of care at the hospital or psychiatric clinic and give feedback to staff.
Clinical teaching or training	Develop the capability of the workforce to manage psychiatric patients.
Risk management and patient safety, for example, morbidity and mortality meetings	Regular morbidity meetings to identify avoidable problems in service delivery.
Implementation of guidelines	To ensure a high standard of care is delivered.
Stakeholder engagement	To ensure better coordination of care between clinics, district or regional hospitals.
Quality improvement cycle	Improve the quality of care provided.

Six appropriate responses from the list below or other relevant responses:

- De-escalation as needed – sedation or restraint (chemical or physical)
- Patients to wear hospital clothes, name tags
- Admit to a ‘safe area’ – secure windows, security of other patients, etc.
- Four hourly ‘head counts’ in the ward to ensure no patients are missing
- Regular patient review
- Refer to the RH after 72 h if still symptomatic
- Outline how patients should be managed if unable to refer to the RH
- Appropriate security personnel

**Further reading**

- Chapter 8: Leadership and Governance. Mash B, editor. Handbook of family medicine. 4th edition. Oxford University Press Southern Africa; 2017.
- KwaZulu-Natal Department of Health. KwaZulu-Natal treatment protocols for mental health disorders [homepage on the Internet] [cited 2024 Mar 01]. Available from: http://www.kznhealth.gov.za/townhill/protocol.pdf
- Darzi AJ, Busse JW, Phillips MR, et al. Guidelines for patient management: Considerations before adoption into practice. Eye. 2022;36:1135–1137. https://doi.org/10.1038/s41433-021-01898-z

### Critical appraisal of research

Read the accompanying article carefully and then answer the following questions. As far as possible, use your own words. Do not copy out chunks from the article. Be guided by the allocation of marks concerning the length of your responses.

- Kaswa R, De Villiers MR. The effect of substance uses on antiretroviral treatment adherence in primary health care. S Afr Fam Pract. 2023;65(2):a5660. https://doi.org/10.4102/safp.v65i1.5660
  1. What was the aim of the study? (2 marks)
  2. Identify three distinct arguments made by the authors to justify and provide a rationale for the study. (5 marks)
  3. Critically appraise the facility sampling strategy. (5 marks)
  4. Critically appraise how the authors address confounding factors in this study. (5 marks)
  5. Critically appraise the measurement of the outcomes of interest. (5 marks)
  6. Define Pearson’s correlation coefficient. (2 marks)
  7. Critically appraise the follow-up of the cohorts. (3 marks)
  8. Explain why a retrospective cohort study would be a challenging design for this question. (3 marks)

**Total: 30 marks**

*Suggested answers:*

**1. What was the aim of the study? (2 marks)**

The researchers aimed to evaluate the effects of substance use on adherence to antiretroviral treatment (ART) among people living with HIV (PLWH) (1 mark) attending primary healthcare facilities in the Mthatha region, Eastern Cape, South Africa. (1 mark)

**2. Identify three distinct arguments made by the authors to justify and provide a rationale for the study. (5 marks)**

Globally, poor adherence to ART is one of the main contributors to virological failure in PLWH. (1) In South Africa, adherence rates are much lower than international standards and targets for adherence, such as the Joint United Nations Programme on HIV/AIDS (UNAIDS) 95-95-95 targets. (1) Recent research showed that low adherence rates are an ongoing challenge in the Eastern Cape which is the province with the third highest prevalence in South Africa. (1) Substance use has been established worldwide as a significant contributor to poor adherence to ARTs in PLWH, as it impacts one’s physical and psychosocial vulnerabilities considerably. (1) Although there is some evidence in sub-Saharan Africa that alcohol use affects adherence to ART treatment, there is no research evidence in the Eastern Cape on the effects of substance use on adherence. (1)

**3. Critically appraise the facility sampling strategy. (5 marks)**

The authors selected two of the largest community health centres covering two-thirds of the sub-district’s primary healthcare needs, which likely contributed to the population’s representativity. (1 mark) However, convenience sampling was used, contributing to sampling bias, as the patients the researchers approached in the two community health centres (CHC’s) may not necessarily represent the complete variety of PLWH who attend the two CHCs. (1 mark) A more representative sample may have been to select patients from randomly selected CHCs and clinics in a way that covered all geographic areas in the region (1 mark) and stratify the sample size contribution of each facility based on its size. (1 mark) The sample size calculation is based on a difference in two proportions, presumably of the adherence measurement. Still, it does not state the difference between the proportions used to assist the reader in grasping its magnitude. (1 mark)

**4. Critically appraise how the authors address confounding factors in this study. (5 marks)**

The researchers considered and assessed the contributions of other factors to poor adherence in this study based on the literature and other differences between the two groups of patients. (1 mark) These include disclosure of HIV status, age, sex, unemployment, socioeconomic status, comorbid medical conditions and duration of follow-up. (1 mark) Bivariate analysis was used to assess these factors’ contributions to the outcomes, and multivariate logistic regression was subsequently used to control for any factors that showed statistically significant associations during the bivariate analysis. (1 mark) However, other possible confounders like mental health disorders are not mentioned or included. (1 mark) Furthermore, the researchers acknowledged that the data collected were self-reported, which may have been under- or over-reported. (1 mark)

**5. Critically appraise the measurement of the outcomes of interest. (5 marks)**

The authors provide clear definitions of adherence in keeping with the international standards and definitions of adherence in the short term (first 6 months). (1 mark) They also use two well-established international but locally validated tools for assessing and measuring adherence. (1 mark) No mention is made of whether the validated tool also included using a translated AIDS Clinical Trial Group questionnaire, especially as isiXhosa is the most prevalent language. (1 mark) However, because the tools are also self-reporting, they continue to have an element of self-reporting bias (1 mark). They are also appropriately elected to define any loss to follow-up as a proxy for nonadherence in both groups. (1 mark)

**6. Define Pearson’s correlation coefficient. (2 marks)**

This is a coefficient used to describe the level of linear association between two variables. (1 mark) It is a number between −1 and 1 where negative values represent a negative correlation, zero represents no correlation and positive numbers mean a positive linear relationship between two variables. (1 mark)

**7. Critically appraise the follow-up of the cohorts. (3 marks)**

The cohorts were assessed simultaneously at baseline and after 6 months by two research assistants at the two primary healthcare facilities (see Figure 1 in Kaswa & De Villiers 2023). (1 mark) A study definition of adherence was reported as following 95% or above the prescribed ART, presumably in line with the UNAIDS 95-95-95 targets although this was not specified. (1 mark) The rationale for the 6-month follow-up (and not a shorter or longer period of follow-up) was not clearly stated by the authors although mention was made in the introduction section of ‘several studies reported that alcohol and other substance used lead to low adherence to ART during the first 6 months of treatment’. (1 mark)

**8. Explain why a retrospective cohort study would be a challenging design for this question. (3 mark)**

A retrospective cohort study requires finding patients who have been established as having poor adherence, likely using proxy measures like virological failure blood results followed by folder reviews for documentation of nonadherence. These definitions would have their validity challenges. (1 mark) Each record would then require reviewing to separate those found to have documented substance use problems and those without substance use, which would also be an exercise vulnerable to reporting and record biases. (1 mark) It would remain difficult to establish causality versus association, given the number of confounding factors mentioned. Alcohol is often a drug of denial and is often under-reported by patients. (1 mark)

### Further reading

- Pather M. Evidence-based family medicine. In Mash B, editor. Handbook of family medicine. 4th ed. Cape Town: Oxford University Press, 2017; p. 430–453.
- The Critical Appraisals Skills Programme (CASP). CASP checklists [homepage on the Internet]. 2023 [cited 2023 Feb 19]. Available from: https://casp-uk.net/casp-tools-checklists/
- Riegelman R. Studying a study and testing a test. 5th ed. Philadelphia, PA: Lippincott Wiliams & Wilkins; 2005.
- Moola S, Munn Z, Tufanaru C, et al. Chapter 7: Systematic reviews of etiology and risk. In: Aromataris E, Munn Z, editors. JBI manual for evidence synthesis [homepage on the Internet]. JBI; 2020 [cited 2023 Feb 19]. Available from: https://synthesismanual.jbi.global

## Objectively structured clinical examination (OSCE) station: Mental health

### The objective of the station

This station tests the candidate’s ability to consult with an adolescent patient presenting with mental health issues.

*Type of station:* Integrated consultation.

*Role players:* Adolescent female and her middle-aged mother

### Instructions for candidate

- You are the family physician working at the consultant clinic of a large DH.
- Your task: Please consult with this patient, who is waiting with her mother to see you.
- You do not need to examine this patient. All examination findings will be provided on request.

### Instructions for the examiner

- This is an integrated consultation station in which the candidate has 20 min.
- Familiarise yourself with the Assessor guidelines, which detail the expected responses from the candidate.
- No marks are allocated. In the mark sheet (Table 2), tick off one of the three responses for each competency listed. Ensure you are clear on the criteria for judging a candidate’s competence in each area.

**TABLE 2:** Marking sheet for consultation station.

Competencies	Candidate’s rating		
	Not competent	Competent	Good
Establishes and maintains a good clinician-patient relationship	-	-	-
Gathering information: history or examination or investigations	-	-	-
Clinical reasoning	-	-	-
Explanation and planning	-	-	-
Management	-	-	-

### Guidance for examiners

- The aim is to establish that the candidate has an effective and safe approach to managing an adolescent patient presenting with mental health issues.
- A working definition of competent performance: the candidate effectively completes the task within the allotted time in a manner that maintains patient safety, even though the execution may not be efficient and well structured.
  ■ *Not competent:* patient safety is compromised (including ethically and legally), or the task is not completed.
  ■ Competent: the task is completed safely and effectively.
  ■ Good: in addition to displaying competence, the task is completed efficiently and in an empathic, patient-centred manner (acknowledges patient’s ideas, beliefs, expectations, concerns or fears).
- It also aims to establish and maintain a good clinician-patient relationship.

The competent candidate is respectful and engages with the patient and her mother in a dignified manner.

*(Ascertains reason for the consultation and makes the patient and her mother feel comfortable while ensuring the ground for confidentiality is set.)*

The good candidate is empathic, compassionate and collaborative, facilitating active participation in key areas of the consultation.

*(Maintains this throughout the consultation.)*

- Gathering information

The competent candidate gathers sufficient information to establish a clinical assessment.

*(Explores mental health, lifestyle, or underlying general medical conditions and screens for diabetes, TB/HIV, syphilis and pregnancy and does a suicide risk assessment.)*

The good candidate additionally has a structured and holistic approach.

(*Explores psychosocial issues – concern about possible underlying medical problems and impact on her education; issues in lifestyle that impact the mental state of the patient; use of drugs or alcohol; concerns, fears of both mother and daughter are explored.)*

- Clinical reasoning

The competent candidate identifies the diagnosis (adolescent depression with *self-harm risk likely triggered by psychosocial stressors*) and acknowledges *the possibility of it being part of a more significant mental health issue such as depression or anxiety.*

The good candidate additionally makes a comprehensive biopsychosocial assessment that may be more biomedically specific (*suspect depression*) and expands on contributory psychosocial issues (*parents’ separation, father moving out, older sibling on drugs, unhealthy lifestyle choices, overeating and lack of physical activity*).

- Explaining and planning

The competent candidate uses clear language to explain to the patient that no clear answer is immediately available but needs further investigations and uses strategies to ensure patient understanding (*questions OR feedback OR reverse summarising*).

The good candidate additionally ensures that the patient and her mother are actively involved in decision-making, paying particular attention to knowledge-sharing and empowerment. She offers the patient a chance for an individual consultation in case she wants to discuss issues without her mother present.

- Management

The competent candidate proposes appropriate intervention (*TSH, HIV, RPR, sleep hygiene trial, consider antidepressants for at least 6 months, explain the importance of adherence and the side effects).*

The good candidate discusses relevant therapeutic *and nonpharmacological interventions (exercise, stress management, psychological counselling, referral to a dietitian) and encourages a family or relationship-oriented approach. Explores how to engage resources at the school, for example, school guidance counsellors and teachers overseeing extracurricular activities.*

### Role play – Instructions for actors

An adolescent female patient and the middle-aged mother of the patient.

Opening statement: ‘Doctor, I have brought in my daughter. I am very worried about her. She has been cutting herself; we need help. I can’t cope’.

The adolescent patient is quiet at this stage and waits for the candidate to engage her and ask her if she is comfortable continuing the consultation with her mother present. She says yes.

Open responses: Freely tell the doctor …:

- You are 16 years old and in Grade 10 at a local high school.
- Been feeling very ‘down’ for the last 3 months.
- You have an urge to cut yourself.
- The cutting does not make you feel better, but you feel that it is something out of your control.
- You have never used drugs or alcohol.

Closed responses: Only tell the doctor if s/he brings this up:

- You have no medical problems that you know of. You have never been admitted to the hospital and have no allergies.
- You have not been sleeping well, affecting your concentration at school.
- Sometimes, you feel angry at the world and take it out on your younger sister, shouting at her for no reason.
- You go to bed but often wake up around 04:00 and cannot go back to sleep.
- Your marks have dropped, and you are not motivated to study. You do not see ‘the point’. You have trouble concentrating at school as you constantly think of everything going on at home and do not sleep well.
- You had a tiny group of friends; two of your friends have transferred to different schools, and you find it challenging to fit in with a new group. Now you prefer to spend most of your time alone.
- You are not in a romantic relationship. You feel no one finds you attractive.
- Daily pattern: school from 07:30 to 15:00. You used to play sports but stopped 3 months ago. Usually, you come straight home and eat, then sleep. You feel tired all the time and have no energy to study. Has several meals throughout the night and noticed you have put on weight over the last 3 months. Most of your clothes are too tight. You spend most of your time on social media platforms, but they make you feel worse about your life; sometimes, this prompts you to cut yourself.
- You have sometimes thought of killing yourself but do not want to add to your mother’s pain. When you cut yourself, you want to feel physical pain.
- You feel hopeless and overwhelmed by the situation in your family and do not see how it will get better.
- On weekends, you spend the day in your room, alternating between eating and sleeping, with very little interaction with your family.
- Family: you are the middle child of three children. Your older brother is 18 years and started abusing drugs 6 months ago. Your younger sister is in Grade 8 and doing exceptionally well at school. Your parents are in the process of separation. Your father moved out of the house 3 months ago. Your mother works 12 h a day, 6 days a week.
- Mother – previously treated for depression a few years back – knows what it feels like, doesn’t want daughter to go through same problems, fears she will take her life.

Feelings: hopelessness, emptiness, unworthiness.

Concerns: you are very concerned about the future of your family unit and whether your mother will be able to cope with the three of you. The mother’s biggest concern is whether her daughter will attempt suicide.

### Clinical findings

General: High body mass index (BMI) adolescent female. Body mass index of 41 kg/m^2^. Well kept. No jaundice, pallor, clubbing, cyanosis, oedema or lymphadenopathy.

Vitals:

- Blood pressure (BP): 135/85 mmHg; peripheral pulse easily palpable, regular rate: 84/min
- Temperature: 36.5 °C

Systemic:

- Skin: superficial parallel cuts over both forearms at different stages of healing
- Respiratory: no abnormalities
- Cardiovascular: no abnormalities
- Neurological: no abnormalities
- Musculoskeletal: no abnormalities
- Urine dipstick: no abnormalities
- Urine pregnancy test: negative
- Mini-Mental State Examination: depressed mood, decreased psychomotor activity, normal speech and thought patterns, good insight, poor judgement.

Side room tests:

- Haemoglobin (Hb): 10.5 g/dL
- Random blood glucose: 5.2 mmol/L

**Further reading**

- De Vries E. How to manage a mental health care user in terms of the Mental Health Care Act. In: Mash B, Brits H, Naidoo M, Ras, T, editors. South African family practice manual. 4th ed. Pretoria: Van Schaik, 2023; pp. 310–317.
- Bauman SE. Primary care psychiatry. A practical guide for Southern Africa. 2nd ed. Cape Town: Juta & Company, 2015; p. 306–329.
- Miller L, Campo JV. Depression in adolescents. NEJM. 2021; 385:445–449. https://doi.org/10.1056/NEJMra2033475

